# Alterações Eletrocardiográficas e do Sistema Nervoso Autônomo com a Mudança de Postura em Crianças e Adolescentes com Distrofia Muscular de Duchenne

**DOI:** 10.36660/abc.20230483

**Published:** 2024-03-21

**Authors:** Rose Mary Ferreira Lisboa da Silva, Nathalia Mussi Monteze, Juliana Gurgel Giannetti, Zilda Maria Alves Meira

**Affiliations:** 1 Universidade Federal de Minas Gerais Faculdade de Medicina Belo Horizonte MG Brasil Universidade Federal de Minas Gerais – Faculdade de Medicina, Belo Horizonte, MG – Brasil; 2 Universidade Federal de Minas Gerais Hospital das Clínicas Belo Horizonte MG Brasil Hospital das Clínicas da Universidade Federal de Minas Gerais – Cardiologia Pediátrica, Belo Horizonte, MG – Brasil

**Keywords:** Distrofia muscular de Duchenne, Eletrocardiografia, Frequência Cardíaca, Sistema Nervoso Autônomo

## Abstract

**Fundamento:**

Distrofia Muscular de Duchenne (DMD) é uma doença neuromuscular hereditária rara. O acometimento cardíaco inicial pode ser assintomático. Portanto, a avaliação por métodos não invasivos pode auxiliar sua abordagem.

**Objetivos:**

Analisar o eletrocardiograma (ECG) e a variabilidade da frequência cardíaca (VFC) do grupo com DMD, e comparar com a do grupo controle pareado por idade.

**Métodos:**

Estudo prospectivo com 27 pacientes masculinos com DMD (idade de 11,9 anos) que foram submetidos à avaliação clínica, ECG, ecocardiograma e Holter. ECG (aumento de 200%) foi avaliado por dois observadores independentes. VFC foi feita no domínio do tempo (24 h) e da frequência na posição supina e sentada. O grupo saudável foi de nove pacientes (11,0 anos). Um valor de p < 0,05 foi considerado estatisticamente significante.

**Resultados:**

A média da fração de ejeção (FE) foi de 60% (34 a 71%). O coeficiente de Kappa para as medidas do ECG variou de 0,64 a 1,00. Foram verificados aumento da relação R/S em V1 em 25,9%, onda Q patológica em 29,6% e QRS fragmentado em 22,2% em regiões inferior/lateral alta, este com correlação negativa com FE (p = 0,006). Houve baixa VFC, sem influência de nenhuma variável, inclusive tratamento. Com a mudança da posição, houve aumento da FC (p = 0,004), porém não houve alteração da VFC. A relação LF/HF foi de 2,7 na DMD e de 0,7 no controle (p = 0,002).

**Conclusões:**

Nos participantes com DMD, as ondas R proeminentes em V1 e alterações nas regiões inferior/lateral alta ocorreram em quase 30% dos casos. Houve menor tônus vagal sem influência das variáveis idade, fração de ejeção, dispersão do QT e tratamento. Apesar do aumento da FC, não houve resposta adequada da VFC com a mudança de posição.

**Figure:**
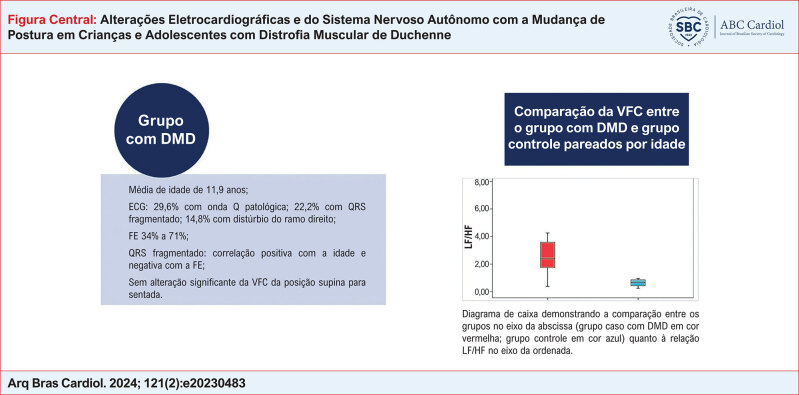
DMD: distrofia muscular de Duchenne; FE: fração de ejeção do ventrículo esquerdo; VFC: variabilidade da frequência cardíaca.

## Introdução

A Distrofia Muscular de Duchenne (DMD) é uma doença neuromuscular hereditária, com início na infância, progressiva, que acomete 1 em 5000 a 6000 nascidos vivos do sexo masculino.^1^ Sua prevalência global é estimada em 4,8 por 100.000 pessoas.^2^ É uma doença genética recessiva ligada ao cromossomo X com mutações no gene da distrofina, resultando em degeneração e necrose muscular, fraqueza muscular, prejuízo da deambulação, comprometimentos comportamental e cognitivo, e miocardiopatia.^1 , 2^

A despeito dos avanços no tratamento, não há cura para a doença. Contudo, a abordagem multidisciplinar permitiu melhora da qualidade de vida e aumento da sobrevida.^1 , 3^ Porém, a miocardiopatia ocorre na maioria dos pacientes e a insuficiência cardíaca é sua principal causa de morte em fase mais tardia, até a terceira década de vida.^1 , 4 , 5^

Em razão da inflamação e fibrose cardíacas,^1^ várias terapias estão em estudo para retardar a progressão da disfunção ventricular, tendo um impacto favorável na sobrevida dos pacientes.^3^ Entretanto, é imperativa a detecção precoce do comprometimento cardíaco. Para atingir essa meta, além da interconsulta cardiológica, uma vez que os pacientes geralmente não apresentam sintomas ou eles são leves em razão de sua doença muscular esquelética, a realização de eletrocardiograma (ECG), exames de imagem como ecocardiograma e/ou ressonância nuclear magnética são indicados.^6^ Biomarcadores emergentes também têm sido pesquisados com o mesmo intuito.^5 , 7^ A função do sistema nervoso autônomo também tem sido avaliada, detectando-se predomínio simpático, o qual é crescente com a progressão da fibrose e, portanto, da doença.^8 – 11^

Entre os medicamentos indicados para prevenir ou reduzir a disfunção sistólica estão os inibidores da enzima de conversão de angiotensina, os bloqueadores do receptor da angiotensina II, o sacubitril, os antagonistas da aldosterona, além dos betabloqueadores (especialmente o carvedilol) e, mais recentemente, a ivabradina.^3 , 6^ As terapias com redução da frequência cardíaca, como o carvedilol e a ivabradina, resultaram em menor proporção de eventos cardíacos de maneira significante.^3^ Apesar disso, do predomínio simpático em pacientes com DMD^8 , 9 , 11^ e da associação entre taquicardia sinusal e progressão para miocardiopatia,^10^ há dois estudos recentes demonstrando que a associação de bisoprolol,^12^ comparando com placebo, ou de carvedilol^13^ com inibidores da enzima de conversão de angiotensina e corticosteroides não apresentou impacto significante no declínio da função cardíaca. Os autores atribuíram esses achados às causas multifatoriais da taquicardia sinusal na DMD, mesmo sem disfunção sistólica, e ao uso de doses moderadas dos betabloqueadores. Portanto, outros estudos, incluindo os randomizados e com maior número de participantes, são necessários para avaliar a dose adequada e de qual betabloqueador entre os aprovados para cardiopatas, além da magnitude da redução da frequência cardíaca e quais pacientes com DMD seriam beneficiados pela terapia precoce com esses bloqueadores adrenérgicos.

Por conseguinte, a estratificação de risco por métodos não invasivos, como ECG e Holter, todavia de baixo custo, pode auxiliar na abordagem dos pacientes com DMD, resultando em início precoce de terapias já estabelecidas, similar ao indicado para aqueles, mesmo assintomáticos, que apresentam realce tardio por meio da ressonância cardíaca.^3^ Desse modo, este estudo teve como objetivos analisar as alterações do ECG e a variabilidade da frequência cardíaca (VFC), inclusive com a mudança de posição, dos participantes com DMD, e comparar essa VFC no domínio da frequência com um grupo controle.

## Métodos

O estudo foi observacional, prospectivo e transversal. A população foi constituída por 27 participantes do sexo masculino com idade entre 5 e 18 anos, com diagnóstico de DMD. Foram excluídos pacientes faltosos às consultas, aqueles com cardiopatia por outra causa, com marca-passo ou prótese valvar, com baixa adesão ao tratamento medicamentoso e em uso de antiarrítmicos, exceto carvedilol. O grupo controle foi composto por nove participantes saudáveis, também do sexo masculino, sem uso de medicação, e pareados por idade aos participantes com DMD. O tamanho da amostra de ambos os grupos foi por conveniência e os participantes eram provenientes do serviço ambulatorial regular, o qual possui 70 pacientes com DMD incluídos em seu cadastro. A pesquisa foi realizada no período de novembro de 2020 a janeiro de 2022.

O projeto de pesquisa foi aprovado pelo Comitê de Ética e Pesquisa da instituição. Todos os participantes e/ou seus responsáveis legais, após convite e esclarecimento, assinaram o termo de assentimento livre esclarecido e/ou o termo de consentimento livre esclarecido.

Os participantes com DMD foram submetidos a avaliação clínica, ECG, Holter digital de 24 h e ecocardiograma transtorácico. O grupo controle foi submetido a avaliação clínica, ECG e Holter.

O ECG foi obtido em 12 derivações, faixa de filtro de 0,16-100 Hz, em papel milimetrado com velocidade de 25 mm/s e amplitude de 10 mm/mV, por meio do programa Windcardio® 11.1.0.0 e avaliado conforme a literatura.^14 , 15^ A dispersão do intervalo QT foi feita considerando-se pelo menos oito derivações. As medidas do ECG (com aumento de 200 vezes pelo aplicativo *Microsoft Paint* ) foram realizadas por dois observadores habilitados e independentes, sem conhecimento das condições clínicas dos participantes, para analisar a variabilidade interobservador, e em duas ocasiões distintas com intervalo de sete dias para analisar a variabilidade intraobservador.

Para a análise da VFC, foi utilizado gravador digital de Holter Cardiolight de 12 derivações, programa CardioSmart CS 550 Versão 6.383, compilação 2.72. A VFC no domínio da frequência (análise espectral) foi feita pela transformação de Fourier. Os participantes permaneceram dez minutos na posição supina, seguida da posição sentada também durante dez minutos, com os registros de todos no final da tarde. Após correção manual de extrassístoles, pausa e interferências, foram obtidos os componentes de alta frequência (HF, *high frequency* ), baixa frequência (LF, *low frequency* ) e a relação entre eles (LF/HF). A partir de registros de 24 h, foram obtidos os índices SDNN, SDANN, SDNNi, rMSSD e pNN50, referentes à VFC no domínio do tempo.^16^ No grupo controle, a análise espectral foi feita somente na posição supina.

### Análise estatística

Para a análise estatística, foi utilizado o programa SPSS ( *Statistical Package for Social Science* ) versão 16.0. Os resultados foram expressos em números e proporções para variáveis categóricas e médias ± desvio-padrão para variáveis contínuas. A confirmação de distribuição normal das variáveis foi verificada pelo teste Shapiro-Wilk. Proporções foram comparadas por meio do teste qui-quadrado ou de Fisher, quando apropriado. O teste t de Student não pareado foi usado para comparar as médias de variáveis de distribuição normal referentes aos participantes com DMD. Para dados quantitativos desses participantes na posição supina e sentada, foi utilizado o teste t de Student pareado para as médias de variáveis de distribuição normal e, para as medianas, foi usado o teste de Wilcoxon. O teste t de Student não pareado foi usado, também, para comparar a média da idade e os componentes da VFC com sua transformação logarítmica entre os grupos caso e controle. Para verificar a concordância intra e interobservador quanto às medidas do ECG, foi utilizada a estatística Kappa. A concordância foi definida como ruim se Kappa inferior a 0,40, como razoável a boa se entre 0,40 e 0,75 e excelente se acima de 0,75. O coeficiente de Pearson foi usado para correlação de variáveis contínuas. Um valor de p < 0,05 foi considerado estatisticamente significante.

## Resultados

A casuística foi composta por 27 participantes com DMD, com média de idade de 11,9 anos. A presença de escoliose foi observada em 12, com média de idade de 15,5 anos. Ao todo, 12 participantes faziam uso de cadeira de rodas, sendo o tempo de uso médio igual a 4,5 ± 2,6 anos (entre 1 e 9 anos). Os demais dados clínicos estão dispostos na Tabela 1 .

**Tabela 1:** Características do grupo com DMD

Variáveis	Média	Desvio padrão	Valor mínimo	Valor máximo
Idade (anos)	11,9	4	6	18
Idade de início de sintomas (anos)	3,7	1,8	1	9
Tempo de diagnóstico (anos)	5,6	1,6	3	9
IMC (kg/m^2^)	21,5	4,9	11,7	30,7
FC (bpm)	99,3	14,9	73	125
PA MSD (mmHg)	101,8/68,5	8,7/7,7	86/50	120/82
PA MSE (mmHg)	99,9/67,8	8,2/7,9	86/50	118/80

IMC: índice de massa corporal; FC: frequência cardíaca na posição supina; bpm: batimentos por minuto; PA: pressão arterial na posição sentada; MSD: membro superior direito; MSE: membro superior esquerdo.

A média da fração de ejeção ao ecocardiograma transtorácico foi de 60,0 ± 0,1%, variando de 34% a 71%. Dezoito participantes com DMD faziam uso de enalapril, 11 de carvedilol, 22 de corticosteroides, quatro de espironolactona e 13 de colecalciferol.

O uso de suporte ventilatório era feito por oito participantes (29,8%), sendo dois em uso de aparelho BIPAP ( *Bilevel Positive Airway Pressure* - pressão positiva nas vias aéreas a dois níveis) e seis em uso de bolsa-válvula-máscara. A média da idade desses participantes foi de 15,5 anos e daqueles sem o suporte ventilatório foi de 10,5 anos (p = 0,002).

A maioria dos participantes com DMD apresentava alteração genética do tipo deleções de éxons (66,7%, n = 18), em 3,7% (n = 1) duplicação de éxon, em 18,5% (n = 5) mutações e em 7,4% (n = 2) códon de parada.

### Achados eletrocardiográficos

**Figura 1:**
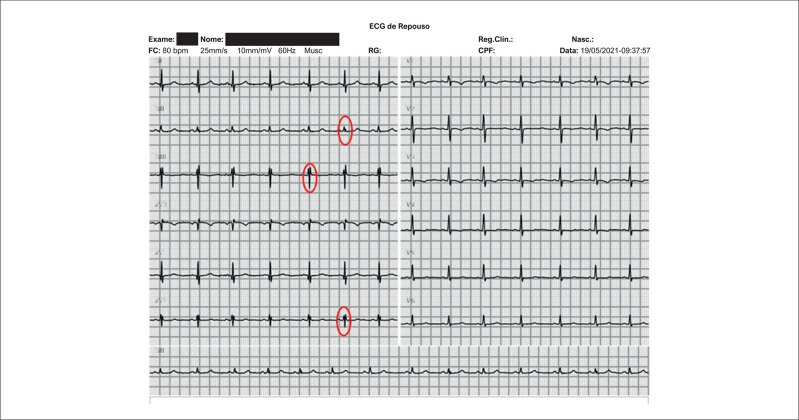
Eletrocardiograma de 12 derivações realizado em um participante de 15 anos com Distrofia Muscular de Duchenne. Os círculos vermelhos indicam deflexão R' com ou sem ondas Q, sem bloqueio de ramo típico (ou seja, duração do QRS < 120ms), em duas derivações contíguas, configurando, assim, a presença de QRS fragmentado em DII, DIII e aVF (parede inferior).

**Figura 2:**
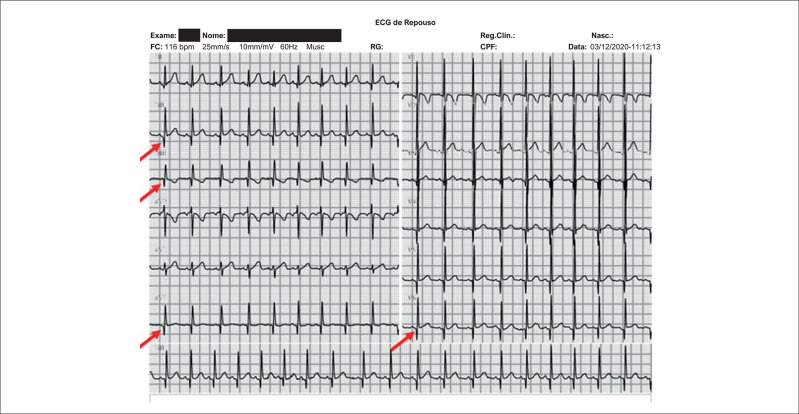
Eletrocardiograma de 12 derivações realizado em um participante de 6 anos com Distrofia Muscular de Duchenne. As setas vermelhas indicam presença de onda Q patológica em DII, DIII e aVF, com amplitudes de 4 a 6 mm.

Por meio do ECG, a média da frequência cardíaca (FC) foi de 101,3 ± 14,7 bpm (variação de 68 a 124 bpm). Em 14,8% (n = 4) foi detectado distúrbio de condução do ramo direito; em 22,2% (n = 6) QRS fragmentado ( Figura 1 ) e em 29,6% (n = 8) presença de onda Q patológica ( Figura 2 ), ambos em regiões inferior e/ou lateral alta.

Em relação às medidas do ECG, as concordâncias pela estatística de Kappa foram de 0,65 (em V2), 0,69 (V1), 0,76 (V3 e V4), 0,88 (D2, aVR e V6), 0,90 (aVL), 0,95 (D1, aVF e V5) e de 1,0 em D3 na análise intraobservador. Na análise interobservador, as concordâncias foram de 0,64 (em aVR), 0,69 (V1 e V2), 0,76 (D2), 0,79 (D1), 0,80 (V3), 0,85 (aVL e aVF), 0,92 (V4), 0,94 (D3) e 0,96 (V5 e V6). Quanto à análise do QRS fragmentado, a concordância apresentou índices entre 0,80 e 0,85.

A média da dispersão do intervalo QT foi de 35,6 ± 11,5 ms, variando de 20 a 60 ms. Esta medida foi obtida em 11 derivações em traçados de cinco participantes e em 12 derivações nos demais. A média do intervalo QT corrigido pela fórmula de Bazett foi de 413,8 ms (de 371,8 a 483,0 ms), pela fórmula de Hodges foi de 393,0 ms (de 356,7 a 444 ms) e pela fórmula de Framingham de 320,8 ms (de 280,1 a 360,1 ms).

Em 25,9% (n = 7) dos pacientes foram observadas ondas R proeminentes e razão R/S aumentada em V1, com média de 1,4 mm (entre 0,11 e 9). Para esta última medida, também realizada por dois observadores, houve excelente concordância interobservador, com coeficiente de Kappa de 0,85.

Houve associação e correlação entre a presença de QRS fragmentado, a idade (15,8 versus 10,8 anos, com e sem o QRS fragmentado, respectivamente, p = 0,005; Pearson de 0,52) e a fração de ejeção (52% versus 63%; p = 0,006, coeficiente de −0,53).

### Análise do sistema nervoso autônomo por meio do Holter

Por meio do Holter, a média da FC foi de 99 ± 9,9 bpm (variando de 77 a 115 bpm). Entre os 17 participantes que apresentaram extrassístoles supraventriculares, a mediana das mesmas foi de 1 [intervalo interquartil Q1-Q3:0-14], máximo de 156, e na maioria (82,3%) o foco era atrial baixo. Entre os 14 participantes que apresentaram extrassístoles ventriculares, a mediana foi de 1 [0-3], com máximo de 1134. A morfologia dessas últimas foi polimórfica em quatro participantes e monomórfica em dez.

**Figura 3:**
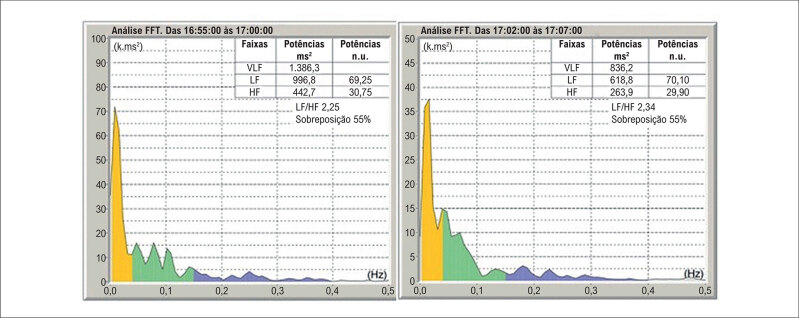
Representação gráfica da análise espectral da frequência cardíaca de participante de 6 anos com DMD na posição supina (A) e na posição sentada (B). VLF: componente de muito baixa frequência (amarelo), LF: componente de baixa frequência (verde) e HF: componente de alta frequência (azul). ms²: metros por segundo ao quadrado; n.u.: unidades normalizadas.

Foi feita a VFC no domínio do tempo, em 24 h, cujos resultados estão demonstrados na Tabela 2 . Estão demonstrados na Tabela 3 os valores da VFC no domínio da frequência, na posição supina e na posição sentada dos participantes com DMD e valores-p por meio dos testes t de Student pareado (para as variáveis de distribuição normal) e de Wilcoxon. A Figura 3 apresenta a análise espectral na posição supina e sentada de um dos participantes com DMD. Não houve diferença significante entre a VFC nos dois domínios e as variáveis idade, fração de ejeção, intervalo e dispersão do QT, e terapia farmacológica.

**Tabela 2:** VFC no domínio do tempo

Variável	Média	Desvio padrão	Mínimo	Máximo
NN médio	615,8	66,5	524,0	782,0
SDNN	97,8	23,6	143,0	86,0
SDANN	82,4	22,9	33,0	125,0
SDNNi	51,2	14,6	23,0	84,0
rMSSD	39,2	17,9	15,0	96,0
pNN50	9,6	7,3	0,5	28,7

NN médio: intervalos de normal a normal médio; SDNN: desvio padrão de todos os intervalos NN; SDANN: desvio padrão das médias dos intervalos NN normais; SDNNi: índice SDNN – média do desvio padrão dos intervalos NN normais; rMSSD: raiz quadrada da média do quadrado das diferenças entre intervalos NN; pNN50: divisão de NN50 (número de diferenças entre intervalos NN sucessivos maiores que 50 ms) pelo total de intervalos NN.

**Tabela 3:** Comparação da VFC no domínio da frequência de pacientes com DMD na posição supina e sentada pelo teste t de Student pareado e pelo teste de Wilcoxon

Variáveis (médias)	Posição supina	Posição sentada	Valor de p
FC (bpm)	101,6 ± 11,2	105,9 ± 14,7	0,004
Potência total	1255,0 * [752,0-2989,0]	1052,5 * [681,7-2876,5]	0,28
VLF (ms^2^)	416,0 * [261,0-1118]	431,0 * [210,0-699,5]	0,56
LF (ms^2^)	482,0 * [306,0-1222,0]	373,5 * [299,2-1069,2]	0,25
HF (ms^2^)	241,0 * [134,0-633,0]	135,0 * [195,0-618,5]	0,73
LF (nu)	67,9 ± 15,1	68,7 ± 14,4	0,54
HF (nu)	32,1 ± 15,1	31,3 ± 14,3	0,57
LF/HF	2,7 ± 1,6	3,0 ± 2,1	0,69

FC: frequência cardíaca; bpm: batimentos/min; VLF: componente de muito baixa frequência; LF: componente de baixa frequência; HF: componente de alta frequência; ms^2^: milissegundos ao quadrado; nu: unidades normalizadas;

* medianas, seguidas do intervalo interquartil [Q1-Q3].

**Figura 4:**
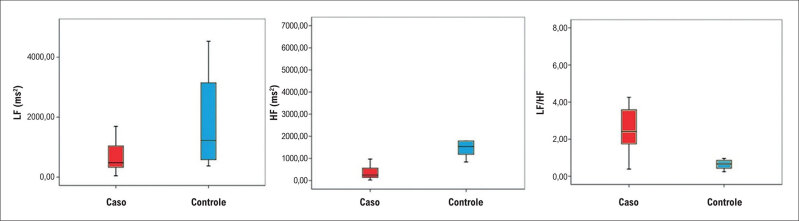
Diagramas de caixas (boxplot) demonstrando a comparação entre os grupos no eixo da abscissa (grupo caso em cor vermelha; grupo controle em cor azul) quanto aos componentes LF (ms²) (A), HF (B) e à relação LF/HF no eixo da ordenada.

Foi realizada, também, a comparação da VFC no domínio da frequência na posição supina do grupo com DMD com a do grupo controle, pareados por idade (média de idade de 12,0 e de 11,0, respectivamente, p = 0,51). Os valores estão demonstrados na Tabela 4 e um exemplo está demonstrado na Figura 4 .

**Table 4:** Valores dos componentes da VFC com transformação logarítmica na posição supina do grupo de estudo comparado ao grupo controle

Variáveis	Grupo caso (n = 27)	Grupo controle (n = 9)	Valor de p
LF (ms^2^)	877,2 ± 896,6	1826,0 ± 1561,5	0,030
HF (ms^2^)	488,7 ± 685,6	2188,5 ± 1820,3	< 0,0001
LF/HF	2,7 ± 1,6	0,7 ± 0,4	0,002

LF: componente de baixa frequência; HF: componente de alta frequência; ms^2^: milissegundos ao quadrado; valor-p com a transformação logarítmica da média dos componentes LF e HF pelo teste t de Student não pareado.

## Discussão

A ausência de distrofina na DMD resulta em degeneração muscular e substituição fibrogordurosa com diminuição da capacidade das células cardíacas, com alterações histológicas, eletrocardiográficas, as quais são precoces, e de imagem. Contudo, há uma variabilidade fenotípica no início e durante a progressão da miocardiopatia. Essa condição pode ser influenciada, também, pela assistência médica.^17 , 18^ A miocardiopatia por DMD pode ser prevista com anos de antecedência, permitindo uma assistência proativa, com uso de medicamentos que evitem o remodelamento ventricular. Entretanto, somente 1/3 dos pacientes com DMD, segundo a literatura, está recebendo esse tratamento.^19^ Portanto, a detecção de alterações precoces, como as eletrocardiográficas e o predomínio simpático antes da disfunção ventricular sistólica, é também determinante para a evolução mais favorável daqueles pacientes.

Neste estudo, os principais achados foram: (1) identificação de ondas Q patológicas e QRS fragmentado, principalmente em parede inferior e/ou lateral alta em até 30% dos participantes com DMD; (2) baixa VFC no domínio do tempo e da frequência no grupo com DMD, detectando-se a presença de disautonomia quando feita a comparação com a análise espectral dos participantes do grupo controle; (3) ausência de alteração significante da VFC com a mudança da posição supina para a sentada, apesar do aumento da FC.

Alterações eletrocardiográficas, como ondas Q em parede inferior, inversões da onda T na parede lateral e bloqueio de ramo direito, podem preceder a disfunção sistólica na DMD e ser detectadas na primeira infância.^20 , 21^ No cenário nacional, em consonância com alguns achados ao ECG do presente estudo, Santos et al.^22^ avaliaram 131 pacientes (média de idade de 9,4 anos) e observaram ondas R anormais em V1 em 29,7%, ondas Q anormais em parede inferior e/ou lateral alta em 37,4%, distúrbio de condução do ramo direito em 55,7% e intervalo QTc prolongado em 35,8% dos pacientes. Uma revisão recente^21^ avaliou estudos com população entre 47 e 246 pacientes com DMD, com idades entre recém-nascido a 41 anos. Não obstante as diferenças entre os estudos, foram observadas ondas Q patológicas em parede inferior e lateral entre 1,6% a 85,7%, aumento da relação R/S na derivação V1, bloqueio de ramo direito entre 0 e 34% dos pacientes, além de taquicardia sinusal. O prolongamento do intervalo QT foi raro. Houve um aumento da incidência de anormalidades no ECG com a idade. As alterações ao ECG foram atribuídas ao comprometimento das células de Purkinje em razão da deficiência da distrofina, ocorrendo antes da inflamação, necrose e fibrose dos cardiomiócitos. Contudo, o aumento da amplitude da onda R em V1 foi atribuído à fibrose da parte basal cardíaca.^21^ Esse achado foi corroborado pelo acometimento mais acentuado das paredes basais inferolaterais e basais inferiores do ventrículo esquerdo por meio do ecocardiograma com análise da deformação miocárdica ( *strain* ) com Doppler tecidual nos pacientes com DMD quando comparados com saudáveis.^23 , 24^

Outra alteração ao ECG foi a presença do QRS fragmentado, resultante da ativação ventricular heterogênea devido a fibrose miocárdica, presente em 22,2% dos pacientes no presente estudo e com moderada correlação positiva com a idade e negativa com a fração de ejeção. Há citação sobre este achado na literatura,^21 , 22^ todavia um estudo com este objetivo foi realizado em 37 pacientes com DMD, com média de idade de 15,6 anos, os quais foram submetidos, também, ao ecocardiograma e à ressonância nuclear magnética. Foi detectado QRS fragmentado em 83,7% dos pacientes, sendo em 76% dos pacientes na parede anterior, 65% na parede lateral e 54% na inferior, ou seja, ocorrendo em mais de uma região. Houve associação dessa fragmentação com disfunção ventricular esquerda, também observada no estudo em questão, e com fibrose e arritmias ventriculares.^25^ Dessa maneira, esse achado de QRS fragmentado pode servir como ferramenta de triagem simples, exequível e de baixo custo na DMD, visto a dificuldade enfrentada por esses pacientes ao serem submetidos a exames de imagem, principalmente à ressonância, em razão de seu acometimento muscular e respiratório.

Outro comprometimento na DMD e que está implicado em sua progressão é a modulação autonômica, com aumento da atividade simpática e diminuição da parassimpática, verificada também em modelos experimentais e em outras distrofias musculares, e com associação com eventos cardiovasculares.^26 , 27^ Assim, alguns estudos têm sido desenvolvidos sobre este tema, com os resultados do presente estudo similares aos da literatura. Yotsukura et al.^28^ demonstraram essas alterações autonômicas com a progressão da DMD em 55 pacientes, incluindo, também, adultos jovens, com diminuição dos componentes vagais (HF, pNN50) e aumento da relação LF/HF. Inoue et al.,^9^ também incluindo pacientes com DMD de até 27 anos, concluíram que o componente SDNN (balanço simpático-vagal) foi mais sensível como índice de disfunção autonômica e sem associação com a função ventricular e com o peptídeo natriurético. Uma metanálise recente^29^ com oito estudos, com casuística entre 17 e 124 pacientes com DMD (total de 549), com idades entre 5 a 44 anos, dois deles sem grupo controle, demonstrou diminuição da atividade vagal por meio dos componentes da VFC no domínio do tempo (SDNN, rMSSD, pNN50) e da frequência (HF), apesar da heterogeneidade dos estudos. Dentre os estudos, o estudo de Dhargave et al.^10^ foi o mais similar ao nosso. Nele, foram incluídas somente crianças com DMD (n = 124), com idade entre 5 e 10 anos, com relação caso-controle de 1:2,5, transversal, demonstrando, também, aumento da relação LF/HF e diminuição do HF e do rMSSD em posição supina.

Para avaliação do barorreflexo com a mudança de posição, foi feita a comparação da VFC pela análise espectral entre a posição supina e a sentada no grupo com DMD. Houve aumento da FC, todavia sem alteração significante da VFC. Em saudáveis, a mudança de postura da supina para o ortostatismo resulta em redução do tônus vagal e ativação simpática pela menor distensão das terminações nervosas dos barorreceptores, com aumento da FC.^30 , 31^ A taquicardia sinusal, inclusive ao repouso, pode ocorrer na DMD sem disfunção sistólica, sendo considerada um risco para miocardiopatia dilatada futura,^9 , 32 , 33^ e pode associar-se ao desequilíbrio simpático vagal.^10^ Inbaraj et al.,^32^ com uma casuística de 38 pacientes com DMD e 37 saudáveis com mediana de idade de 8 anos demonstraram, em concordância com os resultados deste estudo, taquicardia sinusal e diminuição da VFC no grupo com DMD, concluindo que essas alterações podem ser detectadas no estado pré-clínico. Porém, a VFC foi feita somente com a derivação D2, diferente do presente estudo, no qual foram utilizadas 12 derivações e com correção manual de batimentos ectópicos, pausas e interferências.

Na literatura, há somente um estudo sobre VFC com mudança de posição de maneira semelhante ao nosso estudo (da posição supina para sentada), o qual foi realizado com 28 adolescentes com DMD com média de idade de 15,0 anos, detectando-se redução vagal e aumento simpático, ou seja, disautonomia quando os dados foram comparados com o grupo de saudáveis.^34^

Esse desequilíbrio da regulação autonômica na DMD pode ser explicado por fibrose do nó sinusal, hipometabolismo em giros temporais, úncus, hipocampo e cerebelo, alteração da produção de óxido nítrico, além de perda neuronal.^10^ Alterações sinápticas com diminuição de receptores pós-sinápticos do ácido gama-aminobutírico pela deficiência de distrofina no cérebro, como a perda da óxido nítrico-sintase neuronal, também podem explicar aquela disfunção autonômica, assim como os distúrbios cognitivos e de musculatura esquelética.^35 , 36^ Dessa forma, a VFC pode representar uma ferramenta útil para verificar a disautonomia nos pacientes com DMD na fase pré-clínica de acometimento cardíaco, sem associação entre a VFC e a fração de ejeção do ventrículo esquerdo, como demonstrado pelo presente estudo, com impacto favorável na abordagem desses pacientes.

### Limitações

Não foi feita a comparação dos componentes da VFC no domínio da frequência entre os participantes com DMD na posição supina e em ortostatismo para uma avaliação mais adequada do barorreflexo. Entretanto, esses pacientes apresentavam seu comprometimento muscular esquelético e 44,4% deles necessitavam de cadeira de rodas, o que inviabilizou tal avaliação. Não foi realizado o exame de ressonância cardíaca para adequada caracterização tecidual e detecção de presença de fibrose. Além disso, a casuística foi pequena.

## Conclusões

Os participantes com DMD apresentaram onda Q patológica e complexo QRS fragmentado na região inferolateral alta e onda R proeminente em V1 em até 30% dos casos. Houve correlação moderada positiva e negativa entre o QRS fragmentado e a idade e a fração de ejeção, respectivamente. Houve disfunção autonômica com menor tônus vagal sem influência das variáveis idade, fração de ejeção, dispersão do QT e tratamento. Apesar do aumento da FC, não houve resposta adequada da VFC com a mudança de posição.
