# Supplementary material for: Alterações Eletrocardiográficas e do Sistema Nervoso Autônomo com a Mudança de Postura em Crianças e Adolescentes com Distrofia Muscular de Duchenne
Source: Arq Bras Cardiol. 2024 Mar 21;121(2):e20230483. [Article in Portuguese] doi: 10.36660/abc.20230483 (PMC12092046; doi:10.36660/abc.20230483)
Supplement: Translation

# Translation: Electrocardiographic and Autonomic Nervous System Changes after Changes in the Posture of Children and Adolescents with Duchenne Muscular Dystrophy

**Authors:** Rose Mary Ferreira Lisboa da Silva, Nathalia Mussi Monteze, Juliana Gurgel Giannetti, Zilda Maria Alves Meira

**Affiliations:** 1 Belo Horizonte MG Brazil Universidade Federal de Minas Gerais – Faculdade de Medicina, Belo Horizonte, MG – Brazil; 2 Belo Horizonte MG Brazil Hospital das Clínicas da Universidade Federal de Minas Gerais – Cardiologia Pediátrica, Belo Horizonte, MG – Brazil

**Keywords:** Duchenne Muscular Dystrophy, Electrocardiography, Heart Rate, Autonomic Nervous System

## Abstract

**Background:**

Duchenne Muscular Dystrophy (DMD) is a rare inherited neuromuscular disease. At first, cardiac involvement may be asymptomatic. Therefore, assessing patients using non-invasive methods can help detect any changes.

**Objectives:**

Analyze the electrocardiogram (ECG) test and heart rate variability (HRV) of the DMD group and compare the information with that of the age-matched control group.

**Methods:**

A prospective study with 27 male patients with DMD (11.9 years old), who underwent clinical evaluation, ECG, echocardiogram, and Holter monitoring. ECG (200% increase) was assessed by two independent observers. HRV was measured over time (24 h) and in the frequency domain, in the supine and sitting positions. The healthy group consisted of nine patients (11.0 years old). A value of p < 0.05 was considered statistically significant.

**Results:**

The mean ejection fraction (EF) was 60% (34 to 71%). The Kappa coefficient for ECG measurements ranged from 0.64 to 1.00. An increase in the R/S ratio in V1 was observed in 25.9% of the subjects, pathological Q wave in 29.6%, and fragmented QRS in 22.2% in inferior/high lateral regions, with a negative correlation with EF (p = 0.006). There was low HRV, without the influence of any variable, including treatment. With the change in position, there was an increase in HR (p = 0.004), but there was no change in HRV. The LF/HF ratio was 2.7 in the DMD group and 0.7 in the control group (p = 0.002).

**Conclusions:**

In DMD subjects, prominent R waves in V1 and changes in the inferior/high lateral regions occurred in almost 30% of the cases. Lower vagal tone was observed without the influence of the variables age, ejection fraction, QT dispersion, and treatment. Despite the increase in HR, there was no adequate HRV response to the change in position.

## Introduction

Duchenne Muscular Dystrophy (DMD) is a hereditary, progressive, childhood-onset neuromuscular disease that affects 1 in 5,000 to 6,000 live male births.^1^Its global prevalence is estimated at 4.8 per 100,000 people.^2^It is a recessive genetic disease linked to the X chromosome with mutations in the dystrophin gene, resulting in muscle degeneration and necrosis, muscle weakness, impaired walking, behavioral and cognitive impairments, and cardiomyopathy.^1 , 2^

Despite advances in treatment, there is no cure for the disease. Nevertheless, a multidisciplinary approach has allowed better quality of life and increased survival.^1 , 3^Still, most of the patients have cardiomyopathy, and heart failure is the main cause of death later on, happening by the third decade of life.^1 , 4 , 5^

Due to cardiac inflammation and fibrosis,^1^several therapies are being studied to delay the progression of ventricular dysfunction with a favorable impact on patient survival.^3^However, it is fundamental to detect any cardiac involvement early. To achieve this goal, in addition to cardiological consultations, since patients generally do not have symptoms or only have mild symptoms because of their skeletal muscle disease, an electrocardiogram (ECG) and imaging tests, such as echocardiogram and/or MRI, are recommended.^6^Emerging biomarkers have also been researched with the same purpose.^5 , 7^The function of the autonomic nervous system has also been evaluated, detecting sympathetic predominance, which increases with the progression of fibrosis and, therefore, the disease.^8 – 11^

Among the medications indicated to prevent or reduce systolic dysfunction are angiotensin-converting enzyme inhibitors, angiotensin II receptor blockers, sacubitril, aldosterone antagonists, as well as beta-blockers (especially carvedilol) and, more recently, ivabradine.^3 , 6^Therapies that reduce heart rate, such as carvedilol and ivabradine, resulted in a significantly lower proportion of cardiac events.^3^Despite this and the sympathetic predominance in patients with DMD^8 , 9 , 11^and the association between sinus tachycardia and progression to cardiomyopathy,^10^two recent studies showed that the combination of bisoprolol,^12^compared with placebo, or carvedilol^13^with angiotensin-converting enzyme inhibitors and corticosteroids did not have a significant impact on reduced cardiac function. The authors attributed these findings to the multifactorial causes of sinus tachycardia in DMD, even without systolic dysfunction, and to the use of moderate doses of beta-blockers. Therefore, other studies, including randomized ones with a larger number of subjects, are needed to evaluate the appropriate dose and the beta-blocker among those approved for heart disease, in addition to the extent of the reduction in heart rate and which patients with DMD would benefit from an early therapy with these adrenergic blockers.

**Figure:** DMD: Duchenne muscular dystrophy; EF: left ventricular ejection fraction; HRV: heart rate variability.

Consequently, risk stratification using non-invasive methods, such as ECG and Holter monitoring, are cost-effective and can help in the approach of DMD patients, resulting in the early initiation of already established therapies, similar to the therapies recommended for patients with delayed enhancement on cardiac MRI, despite being asymptomatic.^3^On that account, this study analyzed changes in the ECG and heart rate variability (HRV), including after changes in body position, in subjects with DMD, and compared such HRV to that of a control group in the frequency domain.

## Methods

This was an observational, prospective, and cross-sectional study. The population consisted of 27 male subjects aged between 5 and 18 years diagnosed with DMD. Patients who missed appointments were excluded, as well as those with heart disease due to another cause, with a pacemaker or valve prosthesis, with low adherence to drug treatment, and those taking antiarrhythmic drugs, except carvedilol. The control group was made up of nine healthy male subjects who were not taking medication and were matched by age to DMD subjects. The sample size for both groups was determined based on convenience, and the subjects came from the regular outpatient service that has 70 registered DMD patients. The research was carried out from November 2020 to January 2022.

The research project was approved by the institution's Research Ethics Committee. After being invited to participate in the study and have all their questions answered, all subjects and/or their legal guardians signed the informed consent form.

DMD subjects underwent clinical evaluation, ECG, 24-hour digital Holter monitoring, and transthoracic echocardiography. The control group underwent clinical evaluation, ECG, and Holter monitoring.

It was a 12-lead ECG with a filter range of 0.16-100 Hz on graph paper with a speed of 25 mm/s and an amplitude of 10 mm/m, using the Windcardio® 11.1.0.0 software. The tests were evaluated according to the literature.^14 , 15^QT interval dispersion was measured considering at least eight leads. The ECG measurements (with a 200-fold magnification using the Microsoft Paint application) were carried out by two qualified and independent observers, who were blind to the subjects' clinical conditions, to analyze interobserver variability, and on two separate occasions after a seven-day interval to analyze intra-observer variability.

For HRV analysis, a 12-lead Cardiolight digital Holter recorder, CardioSmart CS 550 software, Version 6.383, compilation 2.72, was used. HRV in the frequency domain (spectral analysis) was performed using Fourier transformation. The subjects remained in the supine position for ten minutes and then sat for ten minutes. All values were recorded in the late afternoon. After the manual correction of extrasystoles, pauses, and interferences, high frequency (HF), low frequency (LF), and the ratio between them (LF/HF) were obtained. After 24-hour recordings, the SDNN, SDANN, SDNNi, rMSSD, and pNN50 indices were obtained, referring to HRV in the time domain.^16^In the control group, the spectral analysis was performed only in the supine position.

### Statistical analysis

The SPSS (Statistical Package for Social Science) software was used for statistical analysis, version 16.0. The results were expressed as numbers and proportions for categorical variables and as means ± standard deviation for continuous variables. The Shapiro-Wilk test was used to check the normal distribution of variables. Proportions were compared using the chi-square or Fisher test, where applicable. An unpaired Student's t-test was used to compare the means of normally distributed variables for DMD subjects. For the subjects' quantitative data in the supine and sitting positions, a paired Student's t-test was used for the means of normally distributed variables, and a Wilcoxon test was used for the medians. An unpaired Student's t-test was also used to compare the mean age and HRV components with their logarithmic transformation between the DMD and control groups. A Kappa statistic was used to verify intra- and inter-observer agreement regarding ECG measurements. Agreement was defined as poor if Kappa was below 0.40, as fair to good if Kappa was between 0.40 and 0.75, and as excellent if Kappa was above 0.75. The Pearson coefficient was used to correlate continuous variables. A value of p < 0.05 was considered statistically significant.

## Results

The sample size consisted of 27 DMD subjects, with a mean age of 11.9 years. The presence of scoliosis was observed in 12 subjects, with a mean age of 15.5 years. Twelve subjects relied on a wheelchair, with an average time of use equal to 4.5 ± 2.6 years (between 1 and 9 years). The remaining clinical data are shown in Table 1 .

**Table 1:** Characteristics of the DMD group

Variables	Mean	Standard deviation	Minimum value	Maximum value
Age (years)	11.9	4	6	18
Age at onset of symptoms (years)	3.7	1.8	1	9
Time since the diagnosis (years)	5.6	1.6	3	9
BMI (kg/m^2^)	21.5	4.9	11.7	30.7
HR (bpm)	99.3	14.9	73	125
BP right arm (mmHg)	101.8/68.5	8.7/7.7	86/50	120/82
BP left arm (mmHg)	99.9/67.8	8.2/7.9	86/50	118/80

BMI: body mass index; HR: heart rate in the supine position; bpm: beats per minute; BP: blood pressure in the sitting position.

The mean ejection fraction on transthoracic echocardiography was 60.0 ± 0.1%, ranging from 34% to 71%. Eighteen DMD subjects were taking enalapril, 11 were taking carvedilol, 22 were taking corticosteroids, four were taking spironolactone, and 13 were taking cholecalciferol.

Ventilatory support was required for eight subjects (29.8%), two of whom were using a BIPAP (Bilevel Positive Airway Pressure) device, and six were using a bag-valve mask. The mean age of these subjects was 15.5 years, and the mean age of those without ventilatory support was 10.5 years (p = 0.002).

The majority of DMD subjects had genetic alterations such as exon deletions (66.7%, n = 18), exon duplication (3.7%, n = 1), mutations (18.5%, n = 5), and stop codon (7.4%, n = 2).

### Electrocardiographic Findings

Using the ECG, the mean heart rate (HR) was 101.3 ± 14.7 bpm (range: 68 to 124 bpm). In 14.8% (n = 4) of the subjects, a right bundle branch block was detected; in 22.2% (n = 6), fragmented QRS ( Figure 1 ), and in 29.6% (n = 8), the presence of pathological Q wave ( Figure 2 ), both in the inferior and/or high lateral regions.

**Figure 1:** Twelve-lead electrocardiogram performed on a 15-year-old subject with Duchenne Muscular Dystrophy. Red circles indicate R' deflection with or without Q waves, without typical bundle branch block (i.e., QRS duration < 120 ms), in two contiguous leads, thus confirming the presence of fragmented QRS in DII, DIII, and aVF (inferior wall).

**Figure 2:** Twelve-lead electrocardiogram performed on a 6-year-old subject with Duchenne Muscular Dystrophy. The red arrows indicate the presence of a pathological Q wave in DII, DIII, and aVF, with amplitudes of 4 to 6 mm.

Regarding ECG measurements, the agreements according to the Kappa statistic were 0.65 (V2), 0.69 (V1), 0.76 (V3 and V4), 0.88 (D2, aVR, and V6), 0.90 (aVL), 0.95 (D1, aVF, and V5), and 1.0 (D3) in the intra-observer analysis. In the inter-observer analysis, agreements were 0.64 (aVR), 0.69 (V1 and V2), 0.76 (D2), 0.79 (D1), 0.80 (V3), 0.85 (aVL and aVF), 0.92 (V4), 0.94 (D3), and 0.96 (V5 and V6). Regarding fragmented QRS analysis, the agreement ranged from 0.80 to 0.85.

The mean QT interval dispersion was 35.6 ± 11.5 ms, ranging from 20 to 60 ms. This measurement was obtained with 11 leads in tracings from five subjects, and 12 leads for the others. The mean QT interval corrected by the Bazett formula was 413.8 ms (from 371.8 to 483.0 ms); by the Hodges formula it was 393.0 ms (from 356.7 to 444 ms); and by the Framingham formula, it was 320.8 ms (from 280.1 to 360.1 ms).

Prominent R waves and an increased R/S ratio were observed in V1 in 25.9% (n=7) of the subjects, with a mean of 1.4 mm (from 0.11 to 9). This last measurement, also carried out by two observers, showed excellent interobserver agreement, with a Kappa coefficient of 0.85.

There was an association and correlation between the presence of fragmented QRS and age (15.8 versus 10.8 years, with and without fragmented QRS, respectively, p = 0.005; Pearson 0.52) and ejection fraction (52% versus 63%; p = 0.006, coefficient of −0.53).

### Analysis of the autonomic nervous system using Holter monitoring

Using the Holter, the mean HR was 99 ± 9.9 bpm (ranging from 77 to 115 bpm). Among the 17 subjects who had supraventricular extrasystoles, the median was 1 [interquartile range Q1-Q3:0-14], with a maximum of 156. The majority (82.3%) had a low atrial focus. Among the 14 subjects who had ventricular extrasystoles, the median was 1 [0-3], with a maximum of 1134. Four subjects had polymorphic ventricular extrasystole, and ten had monomorphic ventricular extrasystole.

HRV was measured in the time domain over 24 hours, the results of which are shown in Table 2 . Table 3 shows the HRV values in the frequency domain, with DMD subjects in the supine and sitting positions, and p-values using a paired Student's t-test (for normally distributed variables) and the Wilcoxon test. Figure 3 shows the spectral analysis in the supine and sitting positions of one of the DMD subjects. There was no significant difference between HRV in the two domains and the variables age, ejection fraction, QT interval and dispersion, and pharmacological therapy.

**Table 2:** HRV in the time domain

Variable	Mean	Standard deviation	Minimum	Maximum
Mean NN	615.8	66.5	524.0	782.0
SDNN	97.8	23.6	143.0	86.0
SDANN	82.4	22.9	33.0	125.0
SDNNi	51.2	14.6	23.0	84.0
rMSSD	39.2	17.9	15.0	96.0
pNN50	9.6	7.3	0.5	28.7

Mean NN: mean normal-to-normal intervals; SDNN: standard deviation of all NN intervals; SDANN: standard deviation of the means of normal NN intervals; SDNNi: SDNN index – mean standard deviation of normal NN intervals; rMSSD: square root of the mean of the differences between NN intervals squared; pNN50: division of NN50 (number of differences between successive NN intervals greater than 50 ms) by the total number of NN intervals.

**Table 3:** Comparison of HRV values in the frequency domain, with DMD subjects in the supine and sitting positions, using a paired Student's t-test and the Wilcoxon test

Variables (means)	Supine position	Sitting position	p value
HR (bpm)	101.6 ± 11.2	105.9 ± 14.7	0.004
Total potency	1255.0 * [752.0-2989.0]	1052.5 * [681.7-2876.5]	0.28
VLF (ms^2^)	416.0 * [261.0-1118]	431.0 * [210.0-699.5]	0.56
LF (ms^2^)	482.0 * [306.0-1222.0]	373.5 * [299.2-1069.2]	0.25
HF (ms^2^)	241.0 * [134.0-633.0]	135.0 * [195.0-618.5]	0.73
LF (nu)	67.9 ± 15.1	68.7 ± 14.4	0.54
HF (nu)	32.1 ± 15.1	31.3 ± 14.3	0.57
LF/HF	2.7 ± 1.6	3.0 ± 2.1	0.69

HR: heart rate; bpm: beats/min; VLF: very low-frequency component; LF: low-frequency component; HF: high-frequency component; ms^2^: milliseconds squared; nu: normalized units;

* medians, followed by the interquartile range [Q1-Q3]

**Figure 3:** Graphical representation of the spectral analysis of the heart rate of a 6-year-old DMD subject in the supine position (A) and the sitting position (B). VLF: very low-frequency component (yellow), LF: low-frequency component (green), and HF: high-frequency component (blue). ms^2^: meters per second squared; n.u.: normalized units.

DMD group HRV, in the frequency domain, in the supine position, was also compared to that of the control group, matched by age (mean age of 12.0 and 11.0, respectively, p = 0.51). The values are shown in Table 4 , and an example is shown in Figure 4 .

**Table 4:** HRV values with logarithmic transformation in the supine position - study group vs. control group

Variables	Study group (n = 27)	Control group (n = 9)	p value
LF (ms^2^)	877.2 ± 896.6	1826.0 ± 1561.5	0.030
HF (ms^2^)	488.7 ± 685.6	2188.5 ± 1820.3	< 0.0001
LF/HF	2.7 ± 1.6	0.7 ± 0.4	0.002

LF: low-frequency component; HF: high-frequency component; ms^2^: milliseconds squared; p-value with the logarithmic transformation of the mean of the LF and HF components by the unpaired Student's t-test.

**Figure 4:** Boxplots demonstrating the comparison between the groups on the abscissa axis (study group in red; control group in blue). As for LF (ms^2^) (A), HF (B), and the LF/HF ratio, on the ordinate axis.

## Discussion

The absence of dystrophin in DMD results in muscle degeneration and fibrofatty replacement with decreased capacity of cardiac cells, histological changes, and electrocardiographic changes, which have an early onset and can be detected in imaging tests. Also, phenotypic variability can be observed at the beginning and during the progression of cardiomyopathy, and this condition can also be influenced by medical care.^17 , 18^DMD cardiomyopathy can be predicted years in advance, allowing proactive medical care with the use of medications that prevent ventricular remodeling. However, according to the literature, only 1/3 of the patients with DMD receive such treatment.^19^Therefore, detecting early changes, such as electrocardiographic changes and sympathetic predominance, before systolic ventricular dysfunction, is also critical for a more favorable evolution of the patients.

In this study, the main findings were: (1) identification of pathological Q waves and fragmented QRS, mainly in the inferior and/or high lateral wall, in up to 30% of the DMD subjects; (2) low HRV in the time and frequency domains in the DMD group, detecting the presence of dysautonomia when compared to the control group based on the spectral analysis; (3) no significant change in HRV when changing from the supine to the sitting position, despite increased HR.

Electrocardiographic changes, such as Q waves in the inferior wall, T wave inversions in the lateral wall, and right bundle branch block, may precede systolic dysfunction in DMD and can be detected in early childhood.^20 , 21^In Brazil, in line with some ECG findings in this study, Santos et al.^22^evaluated 131 patients (mean age of 9.4 years) and observed abnormal R waves in V1 in 29.7%, abnormal Q waves in the inferior and/or high lateral wall in 37.4%, right bundle branch conduction disorder in 55.7%, and prolonged QTc interval in 35.8% of the patients. A recent review^21^evaluated studies with a population ranging from 47 to 246 patients with DMD, newborn to 41 years old. Despite the differences between the studies, pathological Q waves in the inferior and lateral walls were observed in 1.6% to 85.7%, increased R/S ratio in the V1 lead, and right bundle branch block from 0 to 34% of the patients, in addition to sinus tachycardia. QT prolongation was rare. There was an increased incidence of ECG abnormalities with age. The ECG changes were attributed to Purkinje cell impairment due to dystrophin deficiency, occurring before inflammation, necrosis, and fibrosis of the cardiomyocytes. However, increased R wave amplitude in V1 was attributed to fibrosis of the basal portion of the heart.^21^This finding was corroborated by the more accentuated involvement of the inferolateral and inferior basal walls of the left ventricle using echocardiography with analysis of myocardial deformation (strain) using tissue Doppler in patients with DMD when compared to healthy subjects.^23 , 24^

Another ECG change was the presence of a fragmented QRS, resulting from heterogeneous ventricular activation due to myocardial fibrosis, present in 22.2% of the patients in this study, with a moderate positive correlation with age and a negative correlation with ejection fraction. This finding is cited in the literature.^21 , 22^Nevertheless, a study with this objective was carried out on 37 patients with DMD, with a mean age of 15.6 years, who also underwent echocardiography and magnetic resonance imaging. Fragmented QRS was detected in 83.7% of the patients, in 76% of the patients in the anterior wall, 65% in the lateral wall, and 54% in the inferior wall, i.e., occurring in more than one region. Such fragmentation was associated with left ventricular dysfunction, also observed in the study in question, and with fibrosis and ventricular arrhythmias.^25^Therefore, fragmented QRS can be used as a simple, feasible, and low-cost screening tool in DMD, considering that DMD patients face great difficulty in undergoing imaging exams, especially MRI, because of their muscular and respiratory involvement.

Autonomic modulation is another DMD symptom that is implicated in disease progression. It involves increased sympathetic activity and decreased parasympathetic activity, also found in experimental models and in other muscular dystrophies, and associated with cardiovascular events.^26 , 27^Thus, some studies have been conducted on the topic, and the results hereof are similar to those found in the literature. Yotsukura et al.^28^demonstrated these autonomic changes with DMD progression in 55 patients, including young adults, with decreased vagal components (HF, pNN50) and increased LF/HF ratio. Inoue et al.,^9^who also included DMD patients up to 27 years of age, concluded that the SDNN component (sympathetic-vagal balance) was more sensitive as an index of autonomic dysfunction, without association with ventricular function and the natriuretic peptide. A recent meta-analysis^29^with eight studies, with a sample size ranging from 17 to 124 patients with DMD (total of 549), aged 5-44 years, two of them without a control group, demonstrated a decrease in vagal activity through HRV components in the time (SDNN, rMSSD, pNN50) and frequency (HF) domains despite the heterogeneity of the studies. Among the studies, the study by Dhargave et al.^10^was the most similar to ours. It included only children with DMD (n = 124), aged between 5 and 10 years, with a study-control ratio of 1:2.5, cross-sectional, also demonstrating increased LF/HF ratio and decreased HF and rMSSD in the supine position.

To evaluate the baroreflex with changes in position, HRV was compared using spectral analysis between the supine and sitting positions in the DMD group. The HR increased, but there was no significant change in HRV. In healthy subjects, changing posture from supine to standing results in reduced vagal tone and sympathetic activation due to the baroreceptor nerve endings being less distended, with an increase in HR.^30 , 31^Sinus tachycardia, even at rest, can occur in DMD without systolic dysfunction, being considered a risk for future dilated cardiomyopathy,^9 , 32 , 33^and can be associated with vagal-sympathetic imbalance.^10^Inbaraj et al.,^32^with a sample size of 38 patients with DMD and 37 healthy subjects with a median age of eight years, demonstrated, in agreement with the results of this study, sinus tachycardia and decreased HRV in the DMD group, concluding that these changes can be detected in the preclinical state. However, HRV was measured only with the D2 lead, unlike the present study that used 12 leads and manually corrected ectopic beats, pauses, and interference.

In the literature, there is only one study on HRV with a change in position in a way similar to our study (from the supine to the sitting position), which was carried out with 28 adolescents with DMD with a mean age of 15.0 years. The study detected a vagal reduction and sympathetic increase, i.e., dysautonomia, when the data were compared to that of the healthy group.^34^

This autonomic regulation imbalance in DMD can be explained by fibrosis of the sinus node, hypometabolism in the temporal gyri, uncus, hippocampus, and cerebellum, altered production of nitric oxide, in addition to neuronal loss.^10^Synaptic changes with a decrease in postsynaptic gamma-aminobutyric acid receptors due to dystrophin deficiency in the brain, such as the loss of neuronal nitric oxide synthase, can also explain the autonomic dysfunction, as well as cognitive and skeletal muscle disorders.^35 , 36^Therefore, HRV may represent a useful tool to verify dysautonomia in patients with DMD in the preclinical phase of cardiac involvement, without association between HRV and left ventricular ejection fraction, as demonstrated by the present study, with a favorable impact on the management of these patients.

### Limitations

A comparison of HRV components in the frequency domain was not made between DMD subjects in the supine and standing positions for a more adequate assessment of the baroreflex. However, these patients had skeletal muscle impairment and 44.4% of them required a wheelchair, which made that assessment unfeasible. A cardiac MRI was not performed for adequate tissue characterization and detection of the presence of fibrosis. Furthermore, the sample size was small.

## Conclusions

DMD subjects had pathological Q waves and fragmented QRS complex in the inferior and high lateral regions and a prominent R wave in V1 in up to 30% of the cases. There was a moderate positive and a negative correlation between fragmented QRS and age and ejection fraction, respectively. Autonomic dysfunction was observed with lower vagal tone without the influence of the variables age, ejection fraction, QT dispersion, and treatment. Despite the increase in HR, there was no adequate HRV response to the change in position.
